# Cloning of *PoAIL6* Gene Related to Somatic Embryogenesis in *Paeonia ostii* ‘Fengdan’

**DOI:** 10.3390/ijms262211006

**Published:** 2025-11-13

**Authors:** Yanting Chang, Xue Zhang, Yayun Deng, Tao Hu, Zehui Jiang, Wenbo Zhang

**Affiliations:** 1 International Center for Bamboo and Rattan, No. 8, Futong Eastern Avenue, Beijing 100102, China; changyanting@icbr.ac.cn (Y.C.); zx949949@126.com (X.Z.); yayundeng@icbr.ac.cn (Y.D.); hutao@icbr.ac.cn (T.H.); jiangzh@icbr.ac.cn (Z.J.); 2 Key Laboratory of National Forestry and Grassland Administration/Beijing for Bamboo & Rattan Science and Technology, No. 8, Futong Eastern Avenue, Beijing 100102, China

**Keywords:** *Paeonia ostii*, *PoAIL6*, somatic embryogenesis, gene clone, subcellular localization

## Abstract

*AINTEGUMENTA-LIKE6* (*AIL6*) is a transcription factor specifically expressed in embryos. It is a key to improving peony regeneration through tissue culture. Based on transcriptomic data from our previous research and the published genome data of *Paeonia ostii,* we identified the *PoAIL6* gene associated with somatic embryogenesis (SE) in *Paeonia ostii* ‘Fengdan’. Structural and phylogenetic analyses were conducted on the *PoAIL6*-encoded protein, and its expression pattern across tissues and embryo developmental stages were explored using real-time quantitative PCR. Our results revealed that *PoAIL6* contained two AP2 conserved domains and the characteristic motif of *AIL6* genes. Phylogenetic analysis revealed that the *PoAIL6* gene has similarity to grape (*Vitis vinifera*) and cocoa (*Theobroma cacao*). *PoAIL6* exhibited the highest expression during early embryonic development, with expression levels gradually decreasing throughout SE progression. It was most highly expressed in peony seeds and showed relatively high expression in callus tissue. This study underscores the pivotal role of *PoAIL6* in the early SE and lays a playground for elucidating its molecular mechanisms, supporting the development of efficient and stable regeneration and transformation systems in peony.

## 1. Introduction

*Paeonia ostii*, a member of the genus *Paeonia* (Paeoniaceae, section Moutan), is a representative woody ornamental species in China, valued for both its aesthetic and medicinal properties [1,2]. China hosts nine peony species, and with the expansion of the peony industry, demand for new varieties with distinctive ornamental traits has increased in domestic and international markets. However, limitations in key ornamental traits and reliance on outdated breeding techniques have become major constraints to industrial development. Additionally, the absence of efficient, stable regeneration and genetic transformation systems has severely hindered progress in targeted molecular breeding and large-scale production. Somatic embryos serve as ideal recipients for efficient propagation and genetic transformation of elite germplasm [3,4].

gSomatic embryogenesis (SE) is asexual reproduction in plants, in which embryos originate from somatic cells instead of gametes [5]. Recent developments have greatly advanced SE research in peony. In *Paeonia ostii* ‘Fengdan’, direct SE and shoot organogenesis have been achieved from mature zygotic embryos, with histological analyses confirming embryo and shoot development [5]. Indirect SE systems enable the utilization of mature embryos for genetic transformation and seedling production in peony [6]. Therefore, elucidating the molecular basis of SE and enhancing embryogenic callus formation are essential for efficient and stable peony regeneration and transformation.

SE is governed by the coordinated expression of genes that control somatic-to-embryogenic cell fate, including WOX, SERK, AGL15, and members of the LEC family [7,8,9,10,11,12,13,14,15,16]. *AIL6* (*PLETHORA3* or *PLT3*), an AP2 subfamily member of the APETALA2/ERF family, is key for embryogenesis, meristem maintenance, and adventitious growth induction [13,17,18,19,20,21,22], regulating cell proliferation during embryo formation and root apical meristem activity [23,24]. Kareem et al. [13] revealed a dual mechanism of PLT proteins in adventitious shoot regeneration. PLT3/5/7 initially activate root stem cell regulators PLT1 and PLT2 to establish pluripotency. *AIL6* also contributes to phyllotaxy stability and lateral root emergence. Together with *ANT* and *AIL7*, *AIL6* regulates shoot apical meristem (SAM) function. In the *ant-ail6-ail7* triple mutant, SAM development ceases, producing only several leaves [25]. Additionally, *AIL6* exhibits partial redundancy in flower development, directly targeting genes such as *LEAFY*, which control polarity establishment, meristem function, flower development, and components of the auxin signaling pathway [21]. Although *AIL6* has been extensively studied in model plants, its function in peony remains largely unexplored.

Based on transcriptomic data of somatic embryos from the published *Paeonia ostii* genome [26,27] and our previous studies [28,29,30], we identified the *PoAIL6* gene associated with SE in *Paeonia ostii* ‘Fengdan’. Structural and phylogenetic analyses were conducted on the *PoAIL6*-encoded protein, and its expression in different tissues and embryo developmental stages in peony was quantified by RT-qPCR. The study aimed to clarify *PoAIL6*’s role in SE and potential involvement in somatic embryo growth and development. Our results lay the groundwork for deciphering how *PoAIL6* controls high-frequency SE in *peony* and offer theoretical support for developing efficient, stable regeneration and transformation systems.

## 2. Results

### 2.1. PoAIL6 Screening and Cloning

A BLAST (BLAST+ 2.13.0) search using the *A*. *thaliana AtAIL6* sequence identified 78 homologous sequences in the transcriptomic database. Among these, 34 harbored two conserved AP2 domains. One candidate showing sequence and an expression pattern similar to *AtAIL6* was identified through sequence alignment, motif analysis, and expression profiling and selected for PCR amplification.

Transcriptomic data from peony somatic embryos at ten developmental stages were analyzed. And the target gene was selected for cloning. The amplified fragment was ligated into a cloning vector, and sequencing confirmed a 100% identity with the expected sequence, successfully cloning the *PoAIL6* gene.

### 2.2. Basic Physicochemical Properties of the PoAIL6 Protein

The PoAIL6 protein comprises 493 amino acids, with an estimated molecular weight of 54.4 kDa. It is predicted to be an unstable, hydrophilic protein (instability index > 40; negative hydrophilicity index). Subcellular localization examination suggests that *PoAIL6* primarily resides in the nucleus (Table 1).

### 2.3. Prediction of PoAIL6’s Secondary Structure, Transmembrane Structure, and Signal Peptide

**Figure 1:**
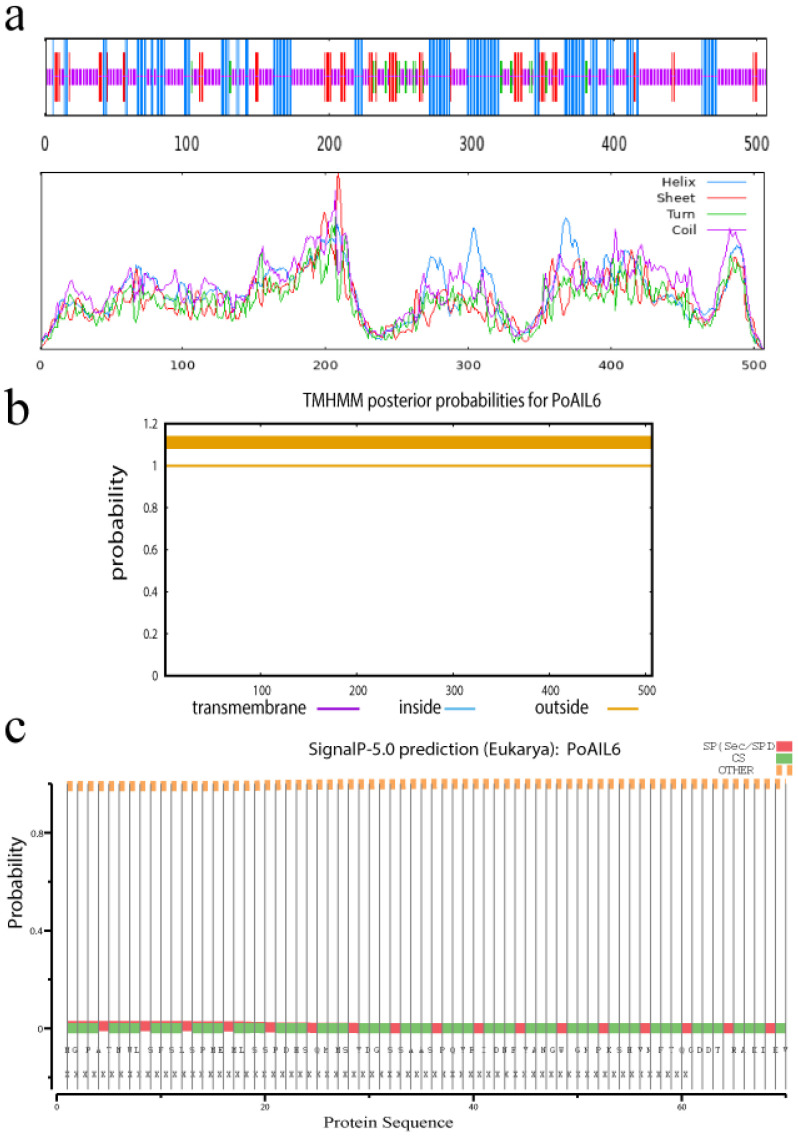
Predicted structure features of *PoAIL6*. (**a**) Secondary structure composition; (**b**) Transmembrane domain distribution; (**c**) Signal peptide presence.

PoAIL6 is predicted to contain 35.23% α-helices, 4.66% β-sheets, 6.74% β-turns, and 53.37% random coils. No transmembrane domains or signal peptides were detected (Figure 1).

### 2.4. Phylogenetic Analysis of the PoAIL6 Amino Acid Sequence

**Figure 2:**
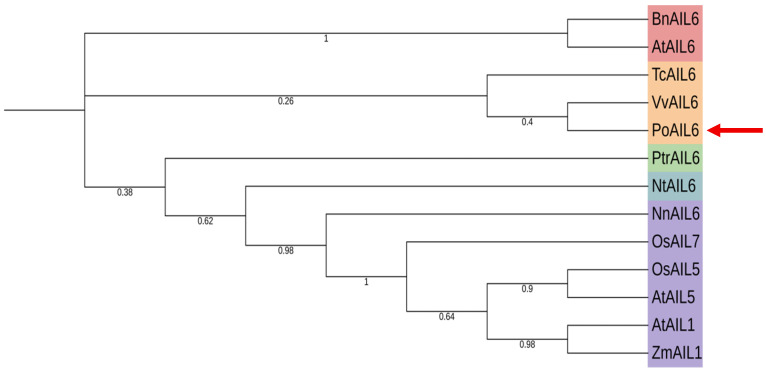
Phylogenetic tree of PoAIL6 and AIL family members from other species. PoAIL6 is indicated by a red triangular arrow.

To explore the evolutionary relationships of *PoAIL6*, a phylogenetic tree was constructed using its protein sequence along with AIL sequences from *A*. *thaliana*, *N*. *tabacum*, *B*. *napus*, *T*. *cacao*, *V*. *vinifera*, *Z*. *mays*, *P*. *trichocarpa*, *O*. *sativa*, and *N*. *nucifera* (Figure 2). The tree agreed with known species relationships. *PoAIL6* clusters with AIL6 homologs, showing the closest relationship to *V*. *vinifera* and *T*. *cacao*.

### 2.5. Conserved Feature and Sequence Alignment of PoAIL6

**Figure 3:**
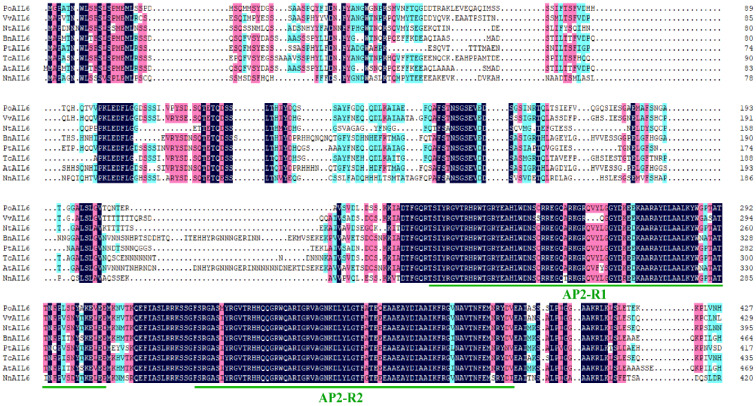
Multiple sequence alignment of PoAIL6 with AIL6 proteins from other species (*A*. *thaliana*, *B*. *napus*, *V*. *vinifera*, *Z*. *mays*, *P*. *trichocarpa*, *T*. *cacao*, and *G*. *max*).

**Figure 4:**
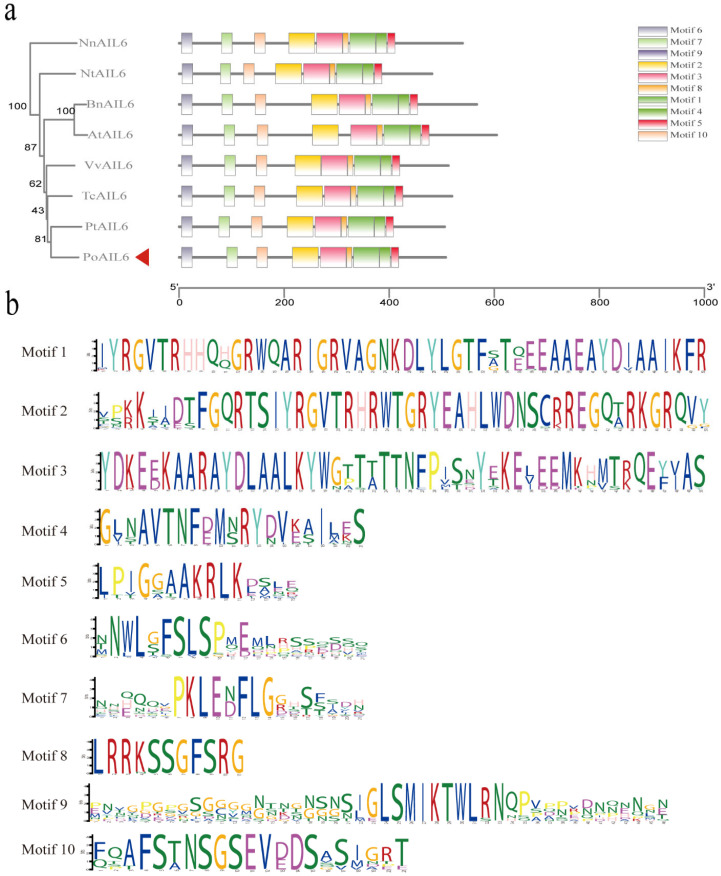
Conserved motif in PoAIL6. (**a**) Phylogenetic tree and conserved motif analysis, with PoAIL6 indicated by a red triangular arrow and the 10 conserved motifs represented by colored square blocks. (**b**) Detailed representation of the 10 conserved motifs.

The AP2 subfamily is divided into the ANT group and the euAP2 group. Based on whether the first AP2/ERF domain and the N-terminal region contain conserved amino acid insertions. The ANT group is divided into euANT and basal ANT. There are eight euANT genes in *Arabidopsis thaliana*: *ANT*, *AIL1*, *AIL2/BBM/PLT4* and *PLETHORAs* (*PLTs*: *AIL3/PLT1*, *AIL4/PLT2*, *AIL6/PLT3*, *AIL5/PLT5*, *AIL7/PTL7*). To evaluate similarity, *PoAIL6* was aligned with AIL6 proteins from eight species (*A*. *thaliana*, *B*. *napus*, *V*. *vinifera*, *Z*. *mays*, *P*. *trichocarpa*, *T*. *cacao*, *N*. *tabacum*, and *N*. *nucifera*). *PoAIL6* harbored AP2-R1 and AP2-R2 domains, characteristic of the AP2 family (Figure 3 and Figure 4), which regulates stem-cell maintenance. It also harbors conserved euANT motifs (euANT1–euANT6, regulate the development of the apical meristem) and an additional histidine insertion within AP2-R2.

### 2.6. Expression Pattern Analysis of the PoAIL6 Gene in Peony

**Figure 5:**
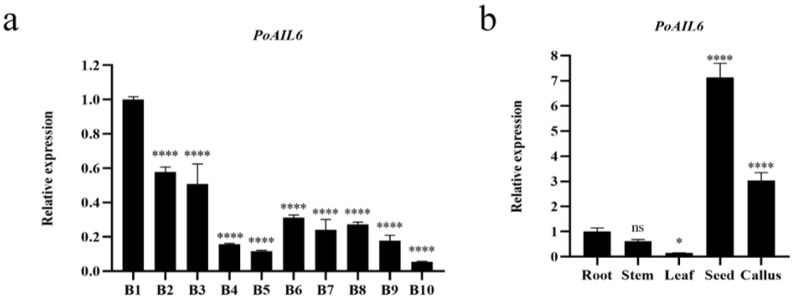
Expression of *PoAIL6* genes in peony using RT-qPCR. (**a**) Expression at ten different timepoints of embryogenic development (0, 5, 10, 15, 20, 30, 40, 60, 70 and 80 days), with 0-day embryos (B1) as the control. (**b**) Expression relative to roots in five tissues. Statistical significance relative to controls is indicated as ns (*p* > 0.05), * (*p* < 0.05), and **** (*p* < 0.0001). Values represent mean ± standard deviation (SD) of three biological replicates, analyzed by one-way ANOVA.

RT-qPCR analysis showed that PoAIL6 expression peaked at stage 1 of somatic embryo development, decreased thereafter, and slightly increased at stages 6–9 (Figure 5). In tissues, PoAIL6 was detected in roots, stems, leaves, seeds, and callus, peaking in seeds and next highest in callus.

### 2.7. PoAIL6’s Subcellular Localization

**Figure 6:**
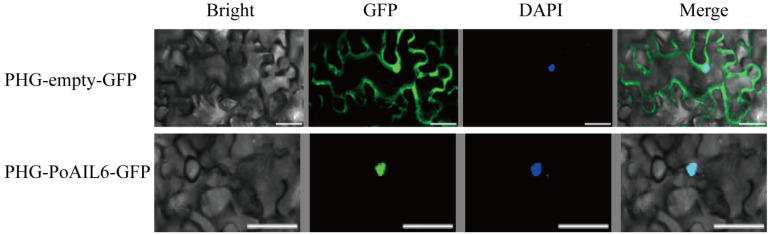
Subcellular localization of PoAIL6. Panels show (from **left** to **right**) bright-field image, fluorescent image of GFP or PoAIL6-GFP fusion protein, DAPI-stained nuclei, and merged image (bright field, GFP, and DAPI). Scale bar = 50 μm.

PoAIL6’s subcellular localization was examined by expressing a PHG-PoAIL6-GFP fusion in *N. benthamiana* leaves, with PHG-empty-GFP as a control. Nuclear-localized GFP signals (Figure 6) matched computational predictions, supporting a role for PoAIL6 as a transcription factor regulating gene expression in the nucleus.

## 3. Discussion

Optimizing a stable and efficient in vitro regeneration system for peony remains a major challenge in genetic breeding. In other woody plants, such as apple, SE-related compounds have been used to enhance regeneration capacity and genetic transformation efficiency. As a “recalcitrant” woody tissue culture species, many embryo regeneration-related genes in peony remain uncharacterized. In this study, an *AIL* gene related to embryo regeneration, *PoAIL6*, was cloned from *P*. *ostia.* This result aligns with previous evidence that *P*. *ostii* (e.g., the “Fengdan” variety) shares an ancestral chromosome duplication event with other core eudicot plants, such as grape [27].

*AILs* genes are considered candidate markers in the SE process and participate in diverse physiological responses and signaling pathways [31,32,33]. However, their specific regulatory mechanisms during somatic embryo formation, cell differentiation, and developmental transitions remain unclear [11,34,35]. Investigating AIL gene expression and regulation in peony SE is therefore critical for enhancing embryo formation and improving regeneration and genetic transformation systems. The high expression of *PoAIL6* in callus tissue further underscores its key role in embryogenesis, suggesting it may promote callus differentiation into buds. Functional redundancy of *PoAIL6* supports the notion that AIL genes are important regulators of early embryo and endosperm development, with considerable potential for SE applications in dicotyledonous plants [36]. Understanding the molecular mechanisms and regulatory networks governing SE is essential for establishing an efficient peony regeneration system. Beyond gene cloning, investigating interactions among proteins encoded by key SE genes is crucial. Subcellular localization experiments showed that PoAIL6 resides in the nucleus, in agreement with its function as a transcription factor and previous studies [15,25]. Similar nuclear localization has been observed for *Platanus acerifolia* PaAIL proteins [37]. Further functional validation of PoAIL6 in peony is needed.

Attempts to transform peony callus using *A. tumefaciens* strains GV3101 and EHA105 encountered challenges, including high contamination rates, suboptimal callus growth after infection, the need for optimization of infection conditions, and strong spontaneous autofluorescence of the callus that interfered with observation. Future work will focus on optimizing the genetic transformation system to improve efficiency and detection accuracy, enabling in-depth exploration of PoAIL6’s role in peony callus development.

## 4. Materials and Methods

### 4.1. Experimental Materials

*Paeonia ostii* ‘Fengdan’ seeds were freshly collected from Heze, Shandong Province, China. Mature zygotic embryos served as explants, first treated in darkness for seven days and then cultured under standard tissue conditions. Samples from somatic embryos were collected at defined stages—0–30 days (cotyledon growth), 40 days (embryo formation), 60 days (secondary embryo germination), 70 days (secondary embryo growth), and 80 days (maturation)—as well as from roots, stems, leaves, seeds, and callus, with three biological replicates per sample, snap-frozen in liquid N2, and kept at −80 °C. Culture was maintained under a 16 h light/8 h dark cycle at 40 μmol·m^−2^·s^−1^ and 23–25 °C in a illumination incubator (MMM, Climacell 404EVO).

SE induction was conducted on Induction medium with MS + 2.0 mg·L^−1^ 2,4-D + 2.0 mg·L^−1^ 6-BA + 30 g·L^−1^ sucrose + 9.2 g·L^−1^ agar. The embryos were cultivated under dark conditions for 20 days, and then they were transferred to a light-dark alternating environment for further cultivation. Proliferation medium was including MS + 0.1 mg·L^−1^ 6-BA + 0.4 g·L^−1^ CH + 60 g·L^−1^ sucrose + 9.2 g·L^−1^ agar, and mature culture medium including MS + 30 g·L^−1^ sucrose + 9.2 g·L^−1^ agar. The pH values of all the above solid culture media were 5.8.

### 4.2. Identification of the PoAIL6 Gene

Based on transcriptomic data from our research group and the published *P*. *ostii* genome data [24,25,26], the *AtAIL6* sequence (*AT5G10510*) from the *Arabidopsis* TAIR database was utilized as a query for local BLAST searches with an E-value set at 1 × 10^−5^. Candidate sequences were preliminarily screened by eliminating duplicates and applying the criteria of identity ≥50% and alignment length ≥ 100 bp. Further screening was performed using HMMER (hmmscan, https://ebi.ac.uk) against the PFAM conserved domain database (https://xfam.org) and the CDD search tools from the National Center for Biotechnology Information (https://www.ncbi.nlm.nih.gov). Genes with two AP2 conserved domains were designated as candidates. Finally, based on transcriptome expression data, the sequence with the highest homology was chosen for subsequent gene amplification.

### 4.3. Protein Multiple Sequence Alignment and Phylogenetic Analysis

*PoAIL6’s* characteristics were predicted using ExPASy-ProtParam (https://web.expasy.org/protparam/) [38]. Its secondary structure was predicted using SOPMA (https://npsa-prabi.ibcp.fr/cgi-bin/npsa_automat.pl?page=npsa_sopma.html) [39]. Transmembrane domains and signal peptides were predicted using TMHMM v.2.0 (https://services.healthtech.dtu.dk/services/TMHMM-2.0/) and SignalP v.6.0 (https://services.healthtech.dtu.dk/services/SignalP-6.0/) [40], respectively. Subcellular localization was inferred using Plant mPLoc (http://www.csbio.sjtu.edu.cn/bioinf/plant-multi/) [41]. Sequences of proteins from other species were downloaded from PlantTFD (https://planttfdb.gao-lab.org) and NCBI (https://www.ncbi.nlm.nih.gov). All web resources were accessed on 24 October 2023. The *PoAIL6* amino acid sequence was aligned with AIL proteins from *Arabidopsis thaliana* (AT5G10510.3), *Brassica napus* (XP_048602759.1), *Vitis vinifera* (RVX23627.1), *Zea mays* (PWZ57740.1), *Populus trichocarpa* (XP_052310498.1), and *Theobroma cacao* (EOX91925.1) using Clustal W v.2.0.11 [42]. Phylogenetic analysis was executed with 1000 bootstrap replicates using the Neighbor-Joining (NJ) method in MEGA 11 [43] and the results were presented using iTOL (https://itol.embl.de).

AIL amino acid sequences from *P*. *ostia, A*. *thaliana*, *B*. *napus*, *G*. *max*, *V*. *vinifera*, *T*. *cacao*, *P*. *trichocarpa*, *Z*. *mays*, *Nicotiana tabacum* (XP_016497688.1), and *Nelumbo nucifera* (XP_019055717.1) were aligned using DNAMAN 9.0 (LynnonBiosoft, San Ramon, CA, USA). Conserved motifs in PoAIL6 and its homologs were identified with MEME (https://meme-suite.org/meme/, accessed 31 October 2023) [4], allowing for up to 10 motifs. The resulting MEME XML and the phylogenetic Newick tree were visualized using the Gene Structure Viewer (Advanced) in TBtools v.2.4.0.119028 [44].

### 4.4. Gene Cloning

RNA was extracted from ten peony embryo stages, five cultured peony seedling tissues using the Huayueyang Rapid Universal Plant RNA Extraction Kit (Tiangen, Beijing, China) and processed into cDNA using the TAKARA Reverse Transcription Kit (TaKaRa, Kusatsu, Japan). Primers were selected using SnapGene (V2.3.2) software (San Diego, CA, USA) (Table 2) based on cDNA sequences. PCR was executed in a 50 µL reaction with 10 µM primers, 5 µL cDNA template, and 25 µL 2× Phanta^®^ Max Master Mix (Vazyme, Nanjing, China) under the following cycling parameters: 98 °C for 8 min, followed by 35 cycles of 98 °C for 1 min, 54 °C for 15 s, and 72 °C for 1 min. Colony PCR was used to identify positive clones, and three were randomly chosen for sequencing by Beijing Azenta Co., Ltd. (Beijing, China).

### 4.5. qRT-PCR

Total RNA was isolated and converted into cDNA, and diluted 10-fold as the template of qPCR with specific primers selected using Primer Premier 6.0 (Table 1).

qRT-PCR was executed on a QTOWER instrument in 10 µL with 1 µL of cDNA template (100 ng/µL), 5 µL of TB Green Premix, and 0.4 µL of each 10 µM primer, with four replicates at conditions of 95 °C for 5 min followed by 45 cycles of 10 s at 95 °C, 15 s at 60 °C, and 15 s at 72 °C. The melting curve was analyzed from 60 to 95 °C, with signals measured at 1 °C increments and data collected every 15 s. *PoAIL6* expression levels relative to the peony ubiquitin gene were calculated using the 2^−ΔΔCt^ method [45], with 0-day somatic embryos and roots as controls.

### 4.6. Subcellular Localization

The cloned CDS was inserted into a PHG vector under a 35S promoter and fused in-frame with GFP. The resulting plasmid 35S::PoAIL6-GFP was delivered into *Agrobacterium tumefaciens* GV3101 competent cells and cultured to an OD600 of ~0.8. The bacterial resuspension was infiltrated into the abaxial epidermis of *N. benthamiana* leaves. After 24 h in the dark, leaves were cultured at a light intensity of at 40 μmol·m^−2^·s^−1^ for 24–48 h. For observation, 0.5–1 cm^2^ of infiltrated leaf tissue was excised, stained with DAPI for 15–20 min in the dark, and rinsed multiple times with sterile water or PBS. DAPI and GFP signals were detected using an Axio Imager M2 laser confocal fluorescence microscope (Zeiss, Oberkochen, Germany).

## 5. Conclusions

The *PoAIL6* gene, associated with SE in *Paeonia ostii* ‘Fengdan’, was cloned and characterized. Structural and phylogenetic analyses were conducted on the encoded protein, and its expression in different tissues and at various embryo developmental stages was analyzed using RT-qPCR. *PoAIL6* likely functions during early SE, providing a basis for elucidating its regulatory mechanisms and enabling optimized peony regeneration and transformation.

## Figures and Tables

**Table 1:** *PoAIL6* gene’s Characteristics.

Physical and Chemical Characteristics	*PoAIL6* Gene
Open reading frame (bp)	1 479
Amino acid number	493
Molecular weight (Da)	54,392.88
Isoelectric point	5.85
II Instability coefficient	45.88
Aliphatic index	61.81
GRAVY	−0.582
Prediction of the subcellular localization	nucleus

**Table 2:** Primers for *PoAIL6* PCR.

Primer Name	Primers Sequence	Application
*PoAIL6*-F	ATGGGTTCTATGAACAACTGGT	CDS amplification primers
*PoAIL6*-R	TTAAGTATCATTCCACACTGTGAAAGT	
Q-*PoAIL6*-F	GGTCCTCTGCTGCTTCTCCTCA	qRT-PCR primers
Q-*PoAIL6*-R	GTTTGGTGTTGGGTGTGGTGGT	

## Data Availability

The original contributions presented in this study are included in the article. Further inquiries can be directed to the corresponding author.
